# Study on the Multi-marker Components Quantitative HPLC Fingerprint of the Compound Chinese Medicine Wuwei Changyanning Granule 

**Published:** 2014

**Authors:** Xian Yang, Shui-Ping Yang, Xue Zhang, Xiao-Dong Yu, Qi-Yi He, Bo-Chu Wang

**Affiliations:** a College of Life Sciences, Chongqing Normal University, Chongqing 401331, P.R.China.; b School of Chinese Materia Medica, Beijing University of Chinese Medicine, Beijing 100029, China.; c College of Resources and Environment, Southwest University, Chongqing, 400716, P.R. China.; d Chongqing Academy of Chinese Materia Medica, Chongqing 400065, P.R.China.; e College of Bioengineering, Chongqing University, Chongqing 400044, PR China.

**Keywords:** The multiple indicator, HPLC fingerprint, Wuwei Changyanning granule, Common peaks number, The percentage of non-common peaks and similarity.

## Abstract

The aim of this paper is to develop a rapid and highly sensitive quantitative HPLC fingerprint method with multiple indicators by using the Compound Chinese Medicine Wuwei Changyanning granule and 5 herbs in the prescription. The quantitative fingerprint chromatogram with multiple indicators was investigated. і)6 compositions included rutin, gallic acid, chlorogenic acid, atractylenolide Ⅰ, pachymic acid and apigenin, which originated from 5 herbs respectively, were selected as quantitative compositions, and their contents were determined using HPLC from 11 batches granules and the corresponding 5 medicinal materials. ⅱ) The precision, stability and repeatability of fingerprinting were investigated. In addition, common peaks number, the percentage of non-common peaks and similarity were also studied. Among them, 21 common peaks in the granule could find the source of peaks from the 5 herbs, among of 10 peaks from Niuerfeng, 9 peaks from Laliao, 3 peaks from Baishu, 3 peaks from Fuling and 5 peaks from Guanghuoxiang. The results showed that the identification method of fingerprinting was reliable.

## Introduction

Traditional Chinese medicines (TCMs) originated from nature, have been widely used in clinical practice (1). Because of its low toxicity and effectively therapeutical performance, TCMs have attracted considerable attention in many ﬁelds. It is well known that the therapeutic effect of the herbal medicine is based on the synergistic effect of their constituents, which makes TCM different from Western medicines (2-3). Quality control, as one of the necessary aspects of TCMs research, is important in guaranteeing the safety, efficacy, and stability of a product. Traditionally, the contents of active components in crude herbs were used to evaluate the quality of the raw plant materials. An herbal medicine may consist of hundreds of compounds, and their contents are variable due to the change of climate, regions of cultivation and seasons of harvest. So TCM is a “black box” system, including numerous unknown compounds that vary greatly in their content and their physical and chemical properties. The complexity of TCMs presents a great challenge for quality control. Fingerprint analysis is now considered an effective method for controlling the quality of TCMs (4). In contrast to other methods, it emphasizes the description of the total characteristics of TCMs, which is appropriate for the characteristics of a “black box” system. Both Food and Drug Administration (FDA) and European Medicines Agency (EMEA) clearly denoted that the appropriate ﬁngerprint chromatogram should be applied to assess the consistency of the botanical drugs(5).Fingerprint analysis in medicinal herbs is an efﬁcient measurement on identifying and assessing the stability of the crude herbs. However, ﬁngerprint analysis only shows the result of similarity calculated based on the relative value with the selected marker compound as reference standard, and does not display the absolute quantity. Obviously, quantitative determination of some marker components is necessary. In this work, chromatographic ﬁngerprint, together with the contents of marker constituents was applied for quality control of TCMs.

Ulcerative Colitis (UC) is an inflammatory bowel disease. Because of slow onset, repeated attack and implied risk of cancerization, and increasing incidence rate year by year, UC has become one of the hot research areas(6). Wuwei Changyanning granule (WWCYNG) is a kind of compound herbal medicine often used to treat UC. The compound medicine comprises five kinds of herbs:* Daphniphullum* *calucinum* Benth. (Niuerfeng), *Polygonum Hydropiper* Linn.(Laliao), *Atractylodes macrocephala* Koidz. (Baishu), *Poria cocos (schw.) *Wolf (Fuling) and *Pogostemon cablin (Blanco) *Benth.(Guanghuoxiang).Rutin is the marker compound came from Niuerfeng, Rutin, gallic acid and chlorogenic acid are the marker compounds for Laliao, atractylenolideⅠ, pachymic acid and apigenin are the marker compounds for Baishu, Fuling and Guanghuoxiang, respectively. 

High performance liquid chromatography (HPLC) is regarded as a prime technique applied to develop ﬁngerprint of crude herbs due to precision, sensitivity and reproducibility (7,8). In this work, we ﬁrstly develop a simple, reliable and reproducible method to establish characteristic ﬁngerprints of Niuerfeng, Laliao, Baishu, Fuling, Guanghuoxiang and their pharmaceutical preparations WWCYNG, and to determine the 6 marker compositions. Both the chromatographic ﬁngerprint and contents of the markers were applied for quality control of TCM.

## Experimental

*Apparatus*

The HPLC apparatus was a SHIMADZU HPLC Pump system (SHIMADZU, Japan) equipped with a photodiode array detector. The column was a SHIMADZU C_18_ column (250 mm×4.6 mm *i.d*., 5 µm). Data acquisition and processing were performed by Empower software.

*Chemicals and materials*

The 6 reference compounds were obtained from the National Institute for the Control of Pharmaceutical and Biological Products (Beijing, China). As shown in Table 1, Niuerfeng, Laliao, Baishu, Fuling and Guanghuoxiang samples were collected from different regions throughout China. Professor Shui-ping Yang (Southwest University, China) identiﬁed all the raw medicinal herbs. 11 batches WWCYNG samples are made by according crude drug samples collected, and also shown in Table 1.Chromatography-grade acetonitrile used for HPLC analysis was purchased from Alltech Scientiﬁc (Beijing, China). HPLC-grade water was obtained from a Milli-Q system (Millipore, Billerica,MA,USA). Methanol, acetic acid, formic acid and phosphoric acid (H_3_PO_4_) were all AR-grade and purchased from the First Chemical Company of Chongqing (Chongqing, China).

**Table1:** Origins of samples collected and WWCYNG

**Sample No.**	**Raw herbal medicine (growth region (China))**					**WWCYNG** ** (Lot No.)**
	Niuerfeng	Laliao	Baishu	Fuling	Guanghuoxiang	
Y_1_	Guangxi	Chongqing	Hunan	Chongqing	Hainan	C_1_
Y_2_	Guangxi	Chongqing	Sichuan	Chongqing	Hainan	C_2_
Y_3_	Guangxi	Chengdu	Zhejiang	Chongqing	Chongqing	C_3_
Y_4_	Chongqing	Hainan	Jiangxi	Hunan	Guangdong	C_4_
Y_5_	Chongqing	Hainan	Yunnan	Hubei	Guangdong	C_5_
Y_6_	Chengdu	Hainan	Chongqing	Yunnan	Guangdong	C_6_
Y_7_	Hainan	Hainan	Chongqing	Guizhou	Guangdong	C_7_
Y_8_	Guangdong	Guangdong	Chongqing	Shanxi	Guangdong	C_8_
Y_9_	Jiangxi	Guangxi	Hubei	Sichuan	Guangdong	C_9_
Y_10_	Jiangxi	Guangxi	Jiangsu	Guangxi	Guangdong	C_10_
Y_11_	Jiangxi	Jiangxi	Guangdong	Anhui	Guangdong	C_11_

Note: 11 batches WWCYNG samples are made by according crude drug samples collected.

*Sample preparation*

Based on the Chinese Pharmacopoeia 2010 edition, 5 crude herbal samples were respectively extracted as follow: Dried and powdered herbal samples about 1.0 g of Niuerfeng and Baishu, 2.0 g of Laliao, Fuling and Guanghuoxiang were respectively transferred to amber ﬂasks and extracted with 50 mL of 80% methanol in an refluxing for 1 h. Additional 80% methanol was added after refluxing to compensate for any lost volume, and the resulting solutions were ﬁltered through a 0.45 µm membrane ﬁlter. A 0.5 g of WWCYNG was transferred to calibrated, amber ﬂasks and extracted with 50 mL of 80% methanol in an ultrasonic bath for 10 min. Additional 80% methanol was added after sonication to compensate for any lost volume, and the resulting solution was ﬁltered through a 0.45 µm membrane ﬁlter.

## Results

*Optimization of the chromatographic conditions*

Optimization of parameters in HPLC was done through investigating the inﬂuence of the detection wavelength, mobile phase, buffer solution and chromatographic column, because these four parameters play a key role on resolution and sensitivity. In this work, we chose a mixture of acetonitrile and water as the mobile phase. Considering the presence of ﬂavonoids in the herbal extraction, a little amount of H_3_PO_4_ was added to the mobile phase to reduce the ionization and lower the polarity of these compounds. The gradient mode was shown as follows: the analysis was carried out on a SHIMADZU ODS column (250 mm×4.6 mm，5 μm). The chromatographic separation was performed with a gradient elution of (A) 0.4% H_3_PO_4_ in water and (B) acetonitrile, 0–8 min, B hold on 9%; 8-20 min, B linear increased from 9 to 14%; 20-40 min，B linear increased from 14 to 20%；40-50 min，B linear increased from 20 to 45%；50-65 min，B linear increased from 45 to 80%；65-88 min，B hold on 80%. The ﬂow rate was set at 0.9mL/min, the column temperature was 30 ℃, injection volume was 10 µL, the wavelengths were selected 273 nm ranging from 0-25 min and 55-88 min, and 360 nm ranging from 25-55 min. 

**Figure 1:**
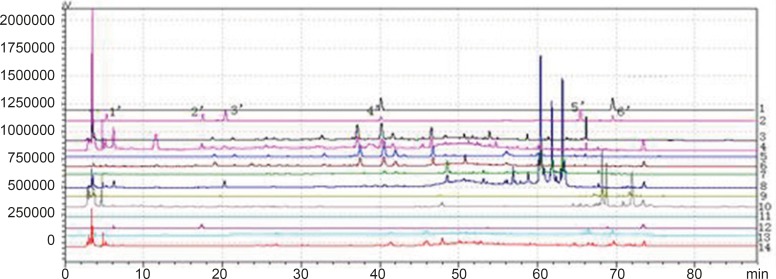
Chromatogram of granule and Chinese herbal medicine in 360 nm and 273 nm Wavelength Note: The number of 1, 3, 5, 7, 9, 11 and 13 were the chromatograms corresponding to mixed standard solution, WWCYNG, Niuerfeng, Laliao, Baishu, Fuling and Guanghuoxiang under 360 nm conditions, respectively. The number of 2, 4, 6, 8, 10 and 12 were the chromatograms corresponding to mixed standard solution, WWCYNG, Niuerfeng, Laliao, Baishu, Fuling and Guanghuoxiang under 273 nm conditions, respectively. The number of 1’, 2’, 3’, 4’, 5’and 6’ were the compents corresponding to Gallic acid (t_R_:5.337 min), Pachymic acid（t_R_:17.386 min), Chlorogenic acid (t_R_:20.314 min), Rutin (t_R_:40.439 min), Atractylenolide Ⅰ (t_R_:65.557 min) and Apigenin ( t_R_:69.453 min), respectively.

*Detection wavelength selection*

**Figure 2:**
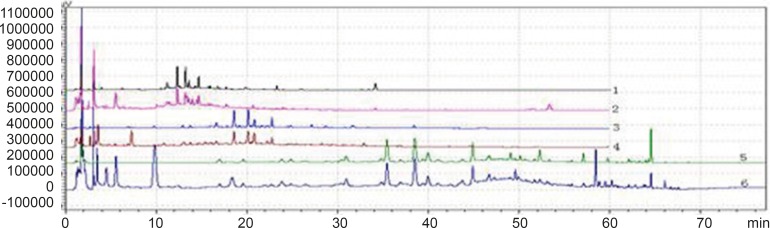
Chromatogram of 3 kinds of gradient elution waies in 360 nm and 273 nm Wavelength (Note: The number of 1,3 and 5 were the WWCYNG chromatograms respectively corresponding to S_I_, S_Ⅱ_ and S_Ⅲ_ under 360 nm conditions. The number of 2, 4 and 6 were the WWCYNG chromatograms respectively corresponding to S_I_, S_Ⅱ_and S_Ⅲ_ under 273 nm conditions

Niuerfeng, Laliao and Guanghuoxiang all contain flavonoids, such as rutin found in Niuerfeng and Laliao, apigenin found in Guanghuoxiang, and their maximum absorption wavelengths are 360 nm or 258 nm, respectively (9). Laliao also contains phenolic acids such as Gallic acid and Chlorogenic acid, which of maximum absorption wavelengths are 273 nm and 327 nm (10). Baishu contains the atractylenolide Ⅰ whose maximum absorption wavelength is 220 nm (11). Fuling contains triterpenoids such as the pachymic acid whose maximum absorption wavelength is 242 nm (12). It is generally known that the ultraviolet spectra is 200-400 nm. Therefore, after other experimental conditions was fixed, we separately selected one low absorption wavelength(273 nm) and one high absorption wavelength (360 nm). The results were shown Figure 2.

As Figure 2 was shown, the detection of wavelength was mainly related with the peak number, peak area of the each peak, the baseline noise and baseline drift, *etc*. ①Peak number: Under 360nm and 273 nm conditions, the chromatogram results of WWCYNG, Niuerfeng, Laliao, Baishu, Fuling and Guanghuoxiang were 39 and 41, 41and 37, 35 and 30, 10and 22, 10and 14, 26 and 29, respectively. ②Peak area: WWCYNG had 2 large chromatographic peaks when the retention time was respectively 11.532 min and 73.442 min in the low wavelength (See The No. 2 in Figure 2), while the 5 herbs had one large chromatographic peak when the retention time was 11.532 min (See The No. 4, 6, 8, 10 and 12 in Figure 2). However, when the wavelength was 360 nm, WWCYNG and the 5 herbs all hadn’t any peak. Therefore, 6 compositions including rutin (Niuerfeng and Laliao), gallic acid (Laliao), chlorogenic acid (Laliao), atractylenolideⅠ(Baishu), pachymic acid (Fuling) and apigenin (Guanghuoxiang), which originated from 5 herbs respectively, were selected as quantitative compositions. The 3 components of gallic acid, pachymic acid and chlorogenic acid almost didn’t appear peak when the detection wavelength was 360 nm, however, they were obvious in the 273 nm UV. Furthermore, the retention time of gallic acid, pachymic acid and chlorogenic acid were 5.337 min, 17.386 min and 20.314 min respectively in the 273 nm UV. So the retention time 25 min was regarded as a switching point，in other words, the detection wavelength was set to 273 nm in 0-25 min. Simultaneously, rutin peak was more obvious in the 360 nm than in the 273 nm, and its retention time was 40.439 min. So the retention time 55 min was regarded as the other switching point. The detection wavelength was set to 360 nm in 25-55 min. 

In summary, this study chose 273 nm and 360 nm as the experimental wavelengths. The elution method was determined among 0-25 min and 55-88 min using 273 nm, but in 25-55 min using 360 nm.

*Mobile phase selection*

The aims of optimization experiment for mobile phase were not only to obtain higher separation efﬁciency and peak resolution of target compounds, but also to reserve shorter run-time. In this work, we chose a mixture of water and acetonitrile as the mobile phase. Considering the presence of ﬂavonoids and triterpenoids in the WWCYNG and herbal extraction, a small amount of H_3_PO_4_ was added to the mobile phase to reduce the ionization and lower the polarity of these compounds. The optimum mobile phase was achieved with A (0.4% H_3_PO_4_+H_2_O) and solvent B (acetonitrile) in the gradient mode achieved after many trials (S_I_ to S_Ⅳ_) shown as in Table 2. The ﬂow-rate was 0.9 mL/min.

**Table 2:** The gradient of mobile phase

**SI**		**S** **Ⅱ**		**S** **Ⅲ**		**S** **Ⅳ**	
**Time/min**	**B%**	**Time/min**	**B%**	**Time/min**	**B%**	**Time/min**	**B%**
0	13	0	13	0	13	0	9
5	13	10	13	5	13	8	9
25	55	35	45	20	25	20	14
50	70	45	45	25	25	40	20
60	90	60	65	45	40	50	45
-	-	-	-	55	60	65	80
-	-	-	-	78	60	88	80

**Figure 3:**
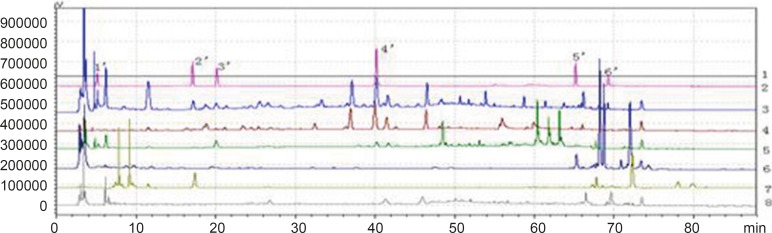
Chromatogram of the S_Ⅳ_ gradient elution way in 360 nm and 273 nm Wavelength

WWCYNG actually was extracted from the 5 herb, so we firstly selected WWCYNG as the research object of mobile phase selection. The chromatograms of SI S_Ⅱ_ and S_Ⅲ_ were merged in one chart, shown in Figure 3.

The result of SI showed：The peak separation of the overall effect from WWCYNG at high wavelengths (See No.1 in Figure 3, and the wavelength monitored at 360 nm) and low wavelengths(See No.2 in Figure 3, and the wavelength monitored at 273 nm) are poor, the peaks are mainly concentrated in the 1.5-10 min and 12-26 min segment. This magnified chromatogram（0-35 min）of granule have a peak in the retention time 6.23 min at 273 nm chromatogram, however there haven’t any peak in the 360 nm chromatograms.

The result of S_Ⅱ_ showed: The chromatographic peaks had the better separation in the range of 10-35 min both 360 nm (See No.3 in Figure 3) and 273 nm (See No.4 in Figure 3). Comparing with the SI whose peaks concentrated in the range of 1 the 1.5-10 min and 12-26 min 0-35 min, the retention time of the corresponding were more reasonable than the retention time of the SI. This magnified chromatogram（0-35 min）of granule can found that the peak number increased more than the SI in the range of 2-10 min at 273 nm chromatogram.

The result of S_Ⅲ_ showed：The total effects of WWCYNG whether peak resolution, peak numbers or baseline drift significantly increased by the select of the S_Ⅲ_ (No.5 in Figure 3 of monitored at 360 nm, No.6 in Figure 3 of monitored at 273 nm). The only weakness is the retention time of first strong peak in 0.638 min, it means first strong peak is too close the position which the solvents peak may also appear, the results lead to the first peak was accurately judged.

On the base the S_Ⅲ_, we selected WWCYNG and 5 herbs as the research object by the selection of mobile phase S_Ⅳ_: Double wavelengths switch mode is shown in Figure 3, by raising the starting proportion of the buffer solution in the mobile phase from 87% to 91%, the retention time of first strong peak changed from 0.638 min to 2.851 min. The results are moderate. Mobile phase S_Ⅳ_ can basically reflects the information about the WWCYNG and herbs, and can be used as the best choice of detection wavelength and mobile phase elution method in this study.

*Quantitative analysis of reference compounds*

The values of RSD of retention times and peak areas were in all cases lower than 1.3%.

*Linearity*

Linearity was examined with standard solutions. A mixed stock solution, containing 12.1 µg/mL Gallic acid, 70.2 µg/mL Pachymic acid, 5.3 µg/mL Chlorogenic acid, 51.6 µg/mL Rutin, 51.0 µg/mL Atractylenolide Ⅰ and 4.8 µg/mL Apigenin ,was prepared. An aliquot (0.1, 0.5, 1.0, 5, 10 and 20 μL) of each standard working solution was subjected to HPLC-DAD analysis. Each calibration curve contained six different concentrations and was performed in triplicate. The linearity for each compound was established by plotting the peak area (y) versus sample quantity (x) of each analyte which was expressed by the equation given in Table 1. All calibration curves showed good linear regression (R^2^>0.999) within test ranges.

*T* *he limit of detection* *(LOD) and the limit of quantification* *(LOQ)*

LOD and LOQ were the concentrations of a compound at which its signal-to-noise ratios (S/N) were detected as 3:1 and 10:1, respectively. They were determined by serial dilution of sample solution using the described HPLC-DAD conditions. The results were shown in Table 3.

*Recovery* *and precision*

Recoveries were performed employing the method of standard addition. Six portions of WWCYNG were spiked with the mixed standards of four alkaloids. Then the samples were pretreated as described in Section 2.3, and the results were summarized in Table 3.All recoveries obtained were very well, indicating the good recovery of the method.

**Table 3:** Linearity and recovery of 6 control compositions

**Compound**	**Linear range** **（** **µg** **）**	**Linear equation**	**Regression r** ^2^ ** (n=5)**	**LOD** **（** **µg/ml** **）**	**LOQ** **（** **µg/mL** **）**	**Recovery**	
						**Average (%)**	**RSD** **（** **%** **）**
Gallic acid,	0.00121-0.242	Y=895909X-255.1	r=0.9998	0.01581	0.05271	100.2	1.21
Pachymic acid	0.00702-1.404	Y=287622X-1876.8	r=0.9993	0.0007197	0.002399	99.85	1.35
Chlorogenic acid	0.00053-0.106	Y=3.0×10^6^X-479.5	r=0.9997	0.0007923	0.02234	100.3	0.78
Rutin	0.00516-1.032	Y=238127X+109.35	r=0.9999	0.009235	0.02671	100.6	0.81
Atractylenolide Ⅰ	0.0051-1.02	Y=558512X+1412.6	r=0.9998	0.00312	0.00994	99.67	0.69
Apigenin	0.00048-0.096	Y=2.0×10^6^X-283.1	r=0.9999	0.00117	0.03562	99.81	1.65

a In the regression equation y=kx+m, y refers to the peak area, x is concentration of the standard substances (µg/mL), r2 is the correlation coefﬁcient of the equation

b Standard deviation is abbreviated as SD.

C LOD (the limit of detection, S/N= 3) and LOQ (limit of quantitation, S/N= 10) were expressed in concentration unit with injection volume 10 µL.

*The* *content determination of* *WWCYNG and compending 5 herbs*

The standard solution and 11 batches herbs of Niuerfeng, Laliao, Baishu, Fuling and Guanghuoxiang, as described in Section 2.3. WCYNG is equivalent and prepared from 2 g Niuerfeng, 1.0 g Laliao, 0.67 g Baishu, 1.0 g Fuling and 0.67 g Guanghuoxiang. 

**Figure 4:**
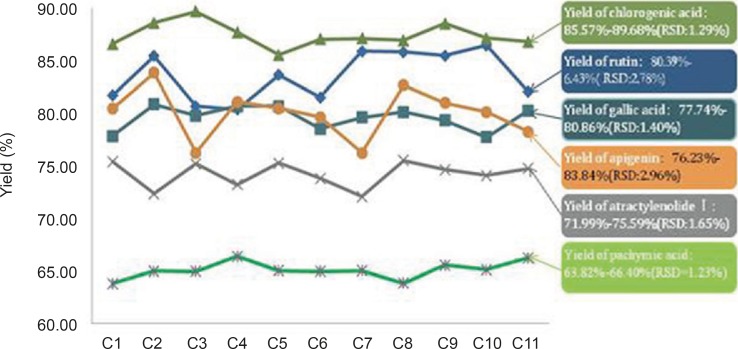
shown, the yield of 11 batches WWCYNG prepared by 5 herbs and the corresponding herbs were as follows:the amount of rutin, gallic acid, chlorogenic acid, atractylenolide Ⅰ, pachymic acid and apigenin were 80.39%-86.43% (RSD=2.78%), 77.74 %-80.86% (RSD=1.40%), 85.57%-89.68% (RSD=1.29%), 71.99 %-75.59% (RSD=1.65%), 63.82 %-66.40%（RSD=1.23%）, 76.23%-83.84% (RSD=2.96%), respectively. R.S.D. values of the yield of from herb to WWCYNG in 11 batches samples were less than 3.0 %, which means the content determinations were in good correspondence in all samples

*Fingerprint analysis of compound herbal medicine* *WWCYNG* *and 5 herbs*

*Common peaks and non-common peaks*

**Figure 5:**
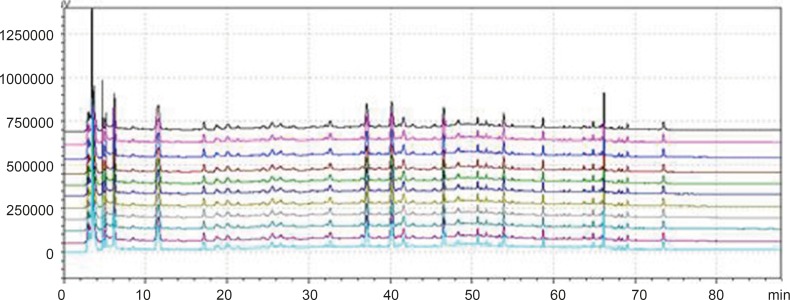
Peak alignment of fingerprints of granule

**Figure 6:**
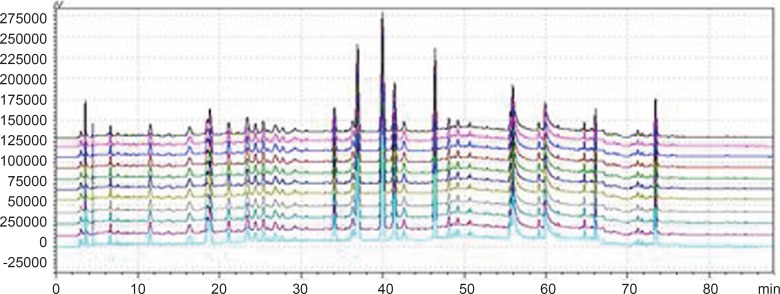
Peak alignment of fingerprints of *Daphniphullum calucinum* Benth

According to the deﬁnition of ﬁngerprints of TCM, a chromatographic ﬁngerprint is in practice a chromatographic pattern of some common kinds of pharmacologically active and chemically characteristic components in the TCM. This chromatographic profile should be characterized by the fundamental attributions of “sameness” and “differences”. It is suggested that the authentication and identiﬁcation of herbal medicines can be accurately conducted by chromatographic ﬁngerprints. The chromatographic ﬁngerprints could demonstrate both the “sameness” and “differences” between various samples successfully (13). With HPLC method, 11 batches of samples from different factories in China were analyzed in the optimum conditions. The average chromatogram from the 11 batches was regarded as the standardized characteristic ﬁngerprint of WWCYNG. Peaks existed in all chromatograms of 11 samples were assigned as “common peaks”, indicating the sameness among various samples. The chromatograms of WWCYNG from the 11 samples consisted of 21 common peaks within 88 min, shown in Figure 5. Among these components, rutin was detected as a high and stable content, therefore it was chosen as the reference substance. All common peaks’ relative retention time and relative peak area were obtained with reference to this substance. R.S.D. values of the relative retention time of 21 common peaks in 11 batches samples were less than 1.0% which means the common peaks were in good correspondence in all samples (data not shown). Moreover, such low R.S.D. values demonstrated that the ﬁngerprint by HPLC had good stability and reproducibility. So, the ﬁngerprint of WWCYNG was composed of the peak proﬁles of the 21 components. Likewise, as shown from Figure 6 to Figure 10,the common peaks of 11 batches about Niuerfeng, Laliao, Baishu, Fuling and Guanghuoxiang, were 19, 15, 12, 7 and 13, respectively.

**Figure 7:**
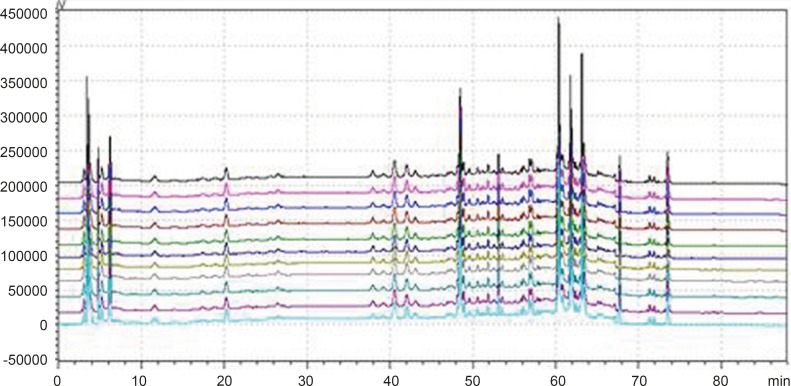
Peak alignment of fingerprints of *Polygonum hydropiper* L.

**Figure 8:**
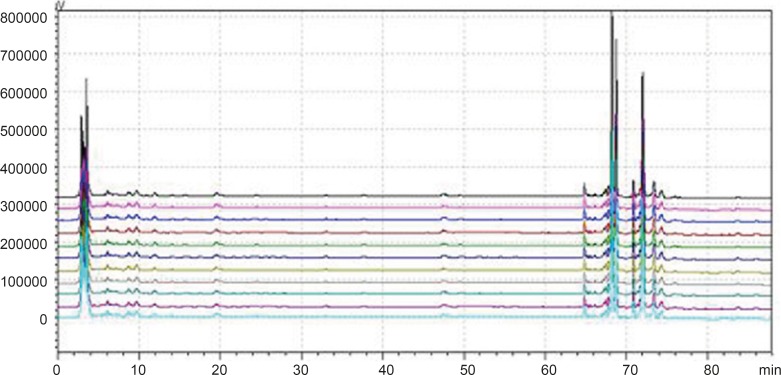
Peak alignment of fingerprints of *Atractylodes macrocephala *Koidz

**Figure 9:**
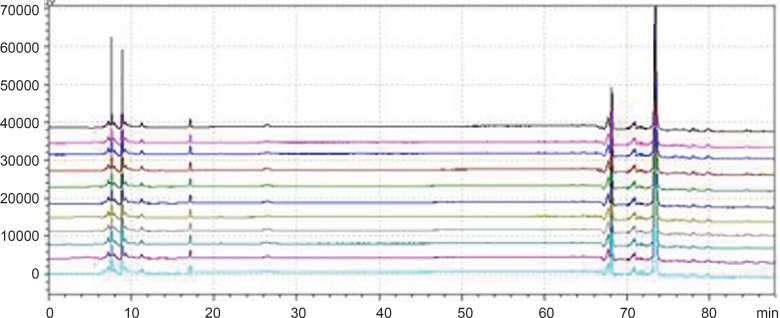
Peak alignment of fingerprints of *Poria cocos*(Schw.)Wolf.

**Figure 10:**
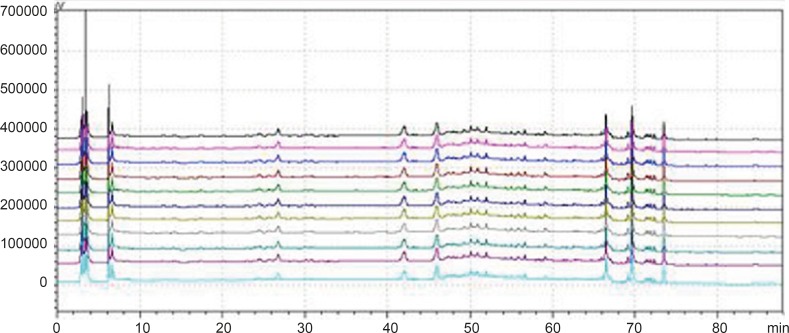
Peak alignment of fingerprints of *Pogostemon cablin*(Blanco)Benth

**Figure 11:**
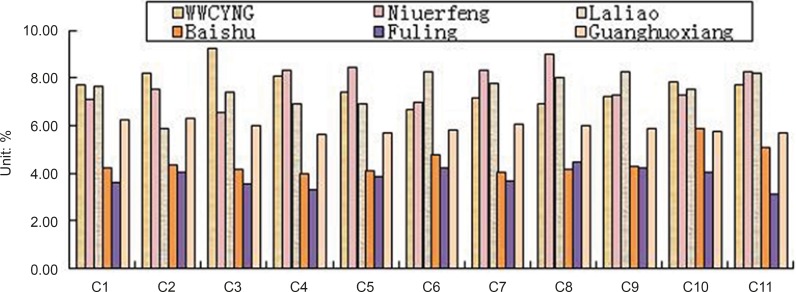
Percentage of unmutual peak area in the total peak area about 11 batches of granules and 5 raw herbs.

Besides the common peaks, there are non-common peaks in each chromatogram, which represents the fuzziness among the same kind of TCM. The percentages of non-common peaks in granules, Niuerfeng, Laliao, Baishu, Fuling and Guanghuoxiang were 6.72% -9.26%, 6.57%-9.01%, 5.88%-8.22%, 3.98%-5.34%, 3.27% -4.44% and 5.70% -6.32%, respectively. They are less than the national standard of 10 % (see Figure 11.).

*Similarity evaluation*

SFDA suggested that all of herbal chromatograms should be evaluated by their similarities, which come from the calculation on the correlation coefﬁcient with the professional software named Similarity Evaluation System for Chromatographic Fingerprint of Traditional Chinese Medicine (Version 2004 A) (14).The ﬁngerprints of 11 batches samples were processed to analyze similarity among these samples. According to the relative peak areas of common peaks, the similarity analysis was conducted (data not shown). The results showed that the similarities of granules, Niuerfeng, Laliao, Baishu, Fuling and Guanghuoxiang are 0.93, 0.93, 0.95, 0.95, 0.94 and 0.92, respectively. All of correlation coefﬁcient of the samples are more than 0.92. These indicated the qualities of WWCYNG and 5 herbs were stable and the products from different pharmaceutical factory were consistent.

*T* *he correlation between WWCYNG preparations and their raw herbal medicines*

The chromatograms of Niuerfeng, Laliao, Baishu, Fuling, Guanghuoxiang and WWCYNG were shown in Table 4. The relative retention time of peaks in WWCYNG ﬁngerprint was compared with that in 5 herbs. It could be found that there were 10, 9, 3, 3 and 5 common peaks in Niuerfeng, Laliao, Baishu, Fuling and Guanghuoxiang, respectively. The correspondence of peaks between in WWCYNG and in raw herbs was shown in details in Table 4. 

**Table 4:** The source analysis of peaks between granule and 5 raw herbs

**Medicines**	**Peak No.**																			
WWCYNG	1	2	3	4	5	6	7	8	9	10	11	12	13	14	15	16	18	19	20	21
Niuerfeng	1	-	-	-	2	3	-	-	9	10	11	12	13	15	-	-	-	-	-	19
Laliao	1	-	3	4	5	-	-	6	-	-	-	7	-	-	-	10	11	-	-	15
Baishu	-	2	-	-	-	-	-	-	-	-	-	-	-	-	-	-	-	7	-	12
Fuling	-	-	-	-	1	-	4	-	-	-	-	-	-	-	-	-	-	-	-	7
Guanghuoxiang	2	-	-	-	3	-	-	-	-	-	-	-	-	-	8	-	-	-	12	13

## Conclusion

A HPLC fingerprint method was established for the quality control of WWCYNG preparations and their raw herbal medicines. The method was well validated by systematically comparing chromatograms of all samples from different region, and certiﬁed helpfully to improve the quality control. Furthermore, the developed method in this study will provide an important reference to establish a quality control method for the traditional Chinese medicinal preparations.
